# The complete chloroplast genome sequence of an Endangered orchid species *Dendrobium bellatulum* (Orchidaceae)

**DOI:** 10.1080/23802359.2018.1437811

**Published:** 2018-02-12

**Authors:** Yun-Jiao Zhang, Cong Ma, Yang Feng, Xing Cheng, Ji Song

**Affiliations:** a Haiyuan College, Kunming Medical University, Kunming, China;; b Yunnan Science Research Institute of Communication & Transportation, Kunming, China;; c College of Ecology and Environmental Sciences, Yunnan University, Kunming, China

**Keywords:** *Dendrobium bellatulum*, chloroplast genome, Convention on International Trade in Endangered Species of Wild Fauna and Flora

## Abstract

*Dendrobium bellatulum* Rolfe is an Endangered orchid species that is distributed in the subtropical regions of Yunnan Province, China. It was listed in the category of the Convention on International Trade in Endangered Species of Wild Fauna and Flora (CITES). Here, we reported the complete chloroplast (cp) genome sequence and the cp genomic features of *D. bellatulum*. The genome was 152,107 bp long with 129 genes comprising 83 protein-coding genes, 40 tRNA genes, and 6 rRNA genes. Phylogenetic analysis of a data set of cp genomes indicated that *D. bellatulum* is clustered with other species in *Dendrobium*.

*Dendrobium bellatulum* Rolfe, is an Endangered orchid species found in subtropical regions of Yunnan Province, China. It is listed in the category of the Convention on International Trade in Endangered Species of Wild Fauna and Flora (CITES). The extensive use of *Dendrobium* materials in healthcare (Halberstein 2005; Shoemaker et al. 2005; Wojcikowski and Gobe 2014) resulted in severe depletion of the wild resources of *Dendrobium*, which has been exploited to near extinction and is now classified as one of the rare and Endangered Orchidaceae plants of China.

Chloroplast genome is much helpful in the molecular identification of *Dendrobium* species (Yukawa et al. 1996; Lau et al. 2001; Zhang et al. 2003; Asahina et al. 2010; Takamiya et al. 2011). In this study, we sequenced the complete chloroplast (cp) genome sequence and reported the cp genomic features of *D. bellatulum*. The specimen of *D. bellatulum* was collected from Yunnan Province of China and stored in Kunming Medical University Haiyuan College. The complete cp-genome sequence was submitted to GenBank under the accession number of MG595965.

Total genomic DNA was extracted from the fresh mature leaves of *D. bellatulum*, and sequenced on Illumina Hiseq 2500 platform (San Diego, CA). Genome sequences were screened out and assembled with CLC genomics workbench as previously reported (Nock et al. 2011), which resulted in a complete circular sequence of 152,107 bp in length. The cp-genome was annotated with Dual Organellar Genome Annotator (DOGMA) (Wyman et al. 2004). The 152,107 bp cp-genome was made up of a large single-copy region (LSC 85,061 bp), a small single-copy region (SSC, 14,503 bp), and two inverted repeat regions (IRs, 26,297 bp). Total GC content is averagely 37.46%, of which the IR regions are 43.0% being a little bit higher than that of the LSC and the SSC regions (35.0% and 30.0%, respectively). A total of 129 genes were successfully annotated, including 89 protein-coding genes, 30 tRNA genes, and 8 rRNA genes. The tRNA genes are distributed throughout the whole genome with 17 in the LSC, 1 in the SSC, and 12 in the IR regions, while rRNAs are only situated in the IR regions. Twelve genes contained one or two introns, nine of which are protein-coding genes. To confirm the phylogenetic position of *D. bellatulum*, a molecular phylogenetic tree was constructed with MEGA 6.0 based on the maximum parsimony method employing a data set of the complete genome sequences of seven species from *Dendrobium*. The results indicated that the *D. bellatulum* is clustered with other species of Orchidaceae (Figure 1). This newly reported chloroplast genome provides a good foundation for the molecular identification of *D. bellatulum*.

**Figure 1.**
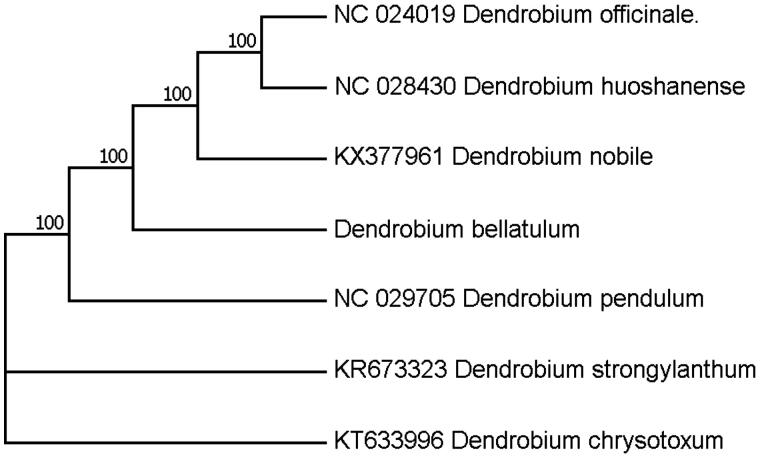
Phylogenetic position of *D. bellatulum* inferred by maximum likelihood (ML) of complete cp genome. The bootstrap values were based on 1000 replicates, and are shown next to the nodes.
